# Prebiotic and Health-Promoting Benefits of Dextran-Type Exopolysaccharide Produced by *Leuconostoc mesenteroides* SJC113

**DOI:** 10.3390/foods14152635

**Published:** 2025-07-27

**Authors:** Dominika Jurášková, Susana C. Ribeiro, Célia C. G. Silva

**Affiliations:** Institute of Agricultural and Environmental Research and Technology (IITAA), University of the Azores, 9700-042 Angra do Heroísmo, Portugal; dominika.juraskova@gmail.com (D.J.); susana.ic.ribeiro@uac.pt (S.C.R.)

**Keywords:** EPS, dextran, prebiotic, lactic acid bacteria, *Leuconostoc mesenteroides*

## Abstract

The exopolysaccharide (EPS) produced by *Leuconostoc mesenteroides* SJC113 is a glucan with α-1,6 and α-3,6 branched glycosidic linkages that may promote human health. The aim of this study was to investigate in vitro the antioxidant, cholesterol-binding, and prebiotic activities of this EPS and its effect on the gut microbiota. The EPS exhibited moderate antioxidant activity, showing free radical scavenging activity (10.94 ± 1.33%) and hydroxyl scavenging activity (6.29 ± 1.59%) at 1 mg/mL. Notably, it showed high cholesterol-binding activity, lowering cholesterol levels by 40% at 1 mg/mL EPS. *Ln. mesenteroides* SJC113 showed strong adhesion to mucin, and its EPS enhanced the adhesion of the probiotic *Lacticaseibacillus rhamnosus* GG. The application of this EPS stimulated the growth of several lactic acid bacteria (LAB) strains in vitro, indicating its potential as a prebiotic. In addition, the use of a human gastrointestinal simulator inoculated with fecal microbiota showed that the EPS favored the growth of *Bifidobacterium* spp. and lactobacilli while reducing *Enterobacteriaceae*. These results emphasize the multifunctional nature of the EPS produced by *Ln. mesenteroides* SJC113 with antioxidant, cholesterol-lowering, and prebiotic properties. Further research is required to investigate the specific mechanisms of action and health benefits in vivo.

## 1. Introduction

The global increase in lifestyle-related diseases emphasizes the need for innovative approaches to promote gut health and overall well-being. Probiotics and prebiotics have proven to be promising strategies to address these challenges [1,2].

Knowledge of the benefits of the gut microbiota in many physiological processes of the host has opened up new possibilities for the use of certain bacterial strains as probiotics. The current scientific evidence suggests that lactic acid bacteria (LAB), particularly lactobacilli (*Bacillota*) and bifidobacteria (*Actinobacteria*), are beneficial to the host in correcting imbalances in the gut microbiota and consequently in maintaining and restoring health [3,4]. Probiotic bacteria can also produce a variety of bioactive compounds, including exopolysaccharides (EPSs). A growing number of studies indicate that certain EPSs produced by LAB have positive physiological effects and serve as fermentable substrates (prebiotics) for the beneficial commensal microbiota [5]. EPSs produced by LAB may also be important for the survival of probiotic bacteria during gastrointestinal transit. EPSs may act as a protective barrier that increases the bacteria’s resistance to harsh conditions, such as low pH, bile salts, and digestive enzymes, thus increasing their survival rate in the gut [6]. In addition, several authors have investigated the effects of prebiotic compounds on the function of probiotics, as these are not broken down during digestion but have a positive effect on the intestinal tract by being utilized by certain groups of bacteria (usually bifidobacteria and lactobacilli) that colonize the intestine [7].

Due to the great differences in their composition and structure, EPSs can have various health effects, such as the control of blood glucose levels, the absorption of calcium and magnesium, as well as antioxidant and anticarcinogenic activities [6,8,9]. Several studies have also shown that EPSs have the potential to influence the gastrointestinal tract, protect intestinal cells from toxins, and lower cholesterol levels [10,11,12].

*Leuconostoc mesenteroides* is a LAB that has attracted attention due to its probiotic properties, in particular, the production of EPSs of the dextran type [13,14]. The structure of these dextrans can vary considerably, affecting their biological activities and functional properties and influencing their solubility and interactions with biological systems [5,6]. Several studies suggest that dextran-type EPSs may play a role in lowering cholesterol levels, potentially contributing to the treatment of dyslipidemia and cardiovascular health [2,7]. The scientific study of dextran-type EPSs is a dynamic area of research with significant implications for health and well-being [4,8]. In a previous study, a strain of *Ln. mesenteroides* (SJC113) isolated from an artisanal cheese was described, which produced a dextran-type EPS composed of glucose with 85% α-(1→6) glycosidic bonds and a small percentage of α-(3→6) bonds [15]. This strain showed good functional and technological properties, and the EPS produced in situ was successfully used as a fat substitute in fresh cheese [15]. The present study addresses the potential of this dextran-like EPS as a novel bioactive compound. Therefore, the specific health-promoting effects of the EPS produced by *Ln. mesenteroides* SJC113 are investigated in this study, focusing on the antioxidant, cholesterol-lowering, and prebiotic effects, as well as on the modulation of the gut microbiota.

## 2. Materials and Methods

### 2.1. Materials

The following materials were used: MRS broth and agar (Biokar Diagnostics, Allonne, France), VRBG agar (Biokar Diagnostics, Allonne, France), PCA agar (Biokar Diagnostics. Allonne, France), peptone water (Biokar Diagnostics, Allonne, France), mucin (Sigma-Aldrich, St. Louis, MO, USA), Trypsin (Sigma, St. Loius, MO, USA), Trichloroacetic acid (PanReac AppliChem ITW reagents, Darmstadt, Germany), DNAse type I (Amresco, Greenville, SC, USA), Pronase E (Amresco, Dallas, TX, USA), DPPH (Sigma, Steinheim, Germany), brilliant green (Sigma, Mumbai, India), L-ascorbic acid (Riedel-de Haën, Seelze, Germany), BHT (Sigma, Madrid, Spain), cholesterol (Sigma-Aldrich, Tokyo, Japan), PBS (Sigma, St. Louis, MO, USA), Fe_2_SO_4_ (Riedel-de Haën, Berlin, Germany), H_2_O_2_ (Panreac Química SAU, Barcelona, Spain), dialysis membrane (10 kDa, Spectrum Laboratories Inc., Piscataway, NJ, USA), sucrose (Sigma, Steinheim, Germany), peptone (Biokar Diagnostics, Allone, France), raffinose (Fluka, Switzerland), lithium chloride (Panreac, Barcelona, Spain) L-cystein (Sigma, Steinheim, Germany), pectin (Condi-Alimentar, Malveira, Portugal), Xylose (Difco, Detroit, MI, USA), Sodium bicarbonate (Sigma, Steinheim, Germany), glucose (Sigma, Lezennes, France), potato starch (Merck, Dermstadt, Germany), yeast extract (Oxoid, Basingstoke, Hampshire, UK), fresh bile (Himedia, Mumbai, India), and Pancreatine (PanReac Applichem, Barcelona, Spain).

### 2.2. Bacterial Strains

Bacterial strains isolated from Azorean cheeses, human feces, and milk, and the commercial probiotic strain *Lacticaseibacillus rhamnosus* GG were used in the present study (Table 1). Stock cultures were kept at −80 °C in 30% (*v*/*v*) glycerol and propagated twice in MRS broth with 1% (*v*/*v*) of inoculum at 37 °C for 24 h.

### 2.3. Production and Purification of EPS

*Ln. mesenteroides* SJC113 was used to produce EPS by incubation of the bacterial culture (1%) in modified MRS broth (10% sucrose) at 30 °C for 48 h. After fermentation, the EPS was extracted according to Jurášková, et al. [15]. Briefly, the medium was centrifuged at 9000× *g* for 5 min, at 4 °C; EPS was precipitated by adding 2 volumes of chilled ethanol, dissolved in Milli-Q water, dialyzed against Milli-Q water at 4 °C for 3 days, and freeze-dried. The phenol–sulfuric acid method was used to quantify EPS yield. Further purification for composition analysis involved DNAse and Pronase E treatment as described by Domingos-Lopes et al. [16].

### 2.4. Biological Activities of EPS

#### 2.4.1. Cholesterol-Binding

The cholesterol-binding ability of EPS was tested by the methodology described by Gawande et al. [17] with some modifications. Purified EPS at 0.01% and 0.1% was resuspended in MRS broth with 3 mg/mL bile salt and 100 µg/mL cholesterol. Uninoculated MRS broth was used as the control. The samples were incubated for 12 h and centrifuged at 10,000× *g* for 20 min. The absorbance of the supernatant was measured at 517 nm and the cholesterol removing ability was calculated as follows:

$$Cholesterol\;removal\;(\%)=\frac{Absorbancecontrol-Absorbancespentbroth}{Absorbancecontrol}\times 100$$

#### 2.4.2. DPPH Scavenging Activity

The effect of scavenging DPPH radicals was measured according to the methodology of Hu et al. [18]. EPS was dissolved in 2 mL of distilled water to obtain 1, 2, and 3 mg/mL. Then, 2 mL of 0.1 mM DPPH was mixed with EPS, incubated in the dark for 30 min, and the absorbance was measured at 517 nm (A_1_). A_2_ was obtained by replacing the DPPH solution with ethanol and A_0_ was a mixture of 2 mL distilled water and 2 mL DPPH solution. Ascorbic acid (0.2–1 mg/mL) was used as a positive control. DPPH scavenging activity was calculated using the following equation:

$$DPPH\;scavenging\;activity\;(\%)=[1-(A_{1}-A_{2})/A_{0}]\times 100$$

#### 2.4.3. Hydroxyl Radical Scavenging Activity

The scavenging activity of hydroxyl radicals was determined using the Fenton’ reaction according to Zhang et al. [19]. For this proposal, 100 µL EPS solutions (1, 2, and 3 mg/mL) were mixed with 0.435 mM brilliant green (100 µL), 0.5 mM FeSO_4_ (200 µL), and 3.0% H_2_O_2_ (150 µL), and incubated for 20 min at room temperature. Before reading the absorbance at 624 nm, the mixture was centrifuged at 5000× *g* for 5 min. The hydroxyl radical scavenging activity was calculated according to the following equation:

$$Hidroxyl\;radical\;scavenging\;activity\;(\%):[(As-A_{0})/(A-A_{0})]\times 100$$

As is the absorbance of the mixture with EPS, A_0_ is the absorbance of a mixture in which the EPS has been replaced by distilled water, and A is the absorbance of the solution obtained by replacing the brilliant green solution with water, labeled A. Ascorbic acid (0.2–1 mg/mL) was used as a positive control.

### 2.5. Prebiotic Properties

#### 2.5.1. Mucin Adhesion Assay

*Ln. mesenteroides* SJC113 was tested for mucin adhesion as described by Dhanani and Bagchi [20]. The probiotic *L. rhamnosus* GG was also tested for comparison. In brief, 96-well plates were coated overnight at 4 °C with 300 µL porcine mucin (0.5 mg/mL in PBS, pH 7.0). The wells with mucin were washed several times with sterile PBS to remove unbound mucin. The overnight bacterial cultures were harvested and washed with PBS. The cell density was adjusted to 1 × 10^8^ CFU/mL in PBS and 200 µL of the bacterial strain was pipetted into each well and allowed to adhere at 37 °C for 90 min. The wells were then washed at least 5 times with PBS to remove all non-adherent cells. To release the adherent cells, 300 µL of Triton X−100 (0.05%) was pipetted into each well and incubated at 37 °C for 20 min. The viable cells were counted after plating on MRS agar and incubation at 37 °C for 48 h.

#### 2.5.2. Effect of EPS on Probiotic Adhesion to Mucin

To evaluate probiotic adhesion in the presence of EPS, the mucin-coated wells were pre-incubated for 15 min at 37 °C with EPS at different concentrations: 0.5, 1, and 2 µg per well. Glucose at a concentration of 1 µg per well was used as a control. After pre-incubation, 200 µL of *L. rhamnosus* GG (1 × 10^8^ CFU/mL) was added to each well and incubated at 37 °C for 90 min. The steps were then repeated as previously described (Section 2.5.1).

#### 2.5.3. Stimulation of LAB Growth

To investigate the effect of EPS isolated from *Ln. mesenteroides* SJC113 on LAB growth, the bacterial cells were grown in MRS (0.5% glucose) as a control and in a 0.5% glucose with MRS (0.5% glucose) supplemented with 1% EPS, according to Liu et al. [21]. The LAB strains used were *L. lactis* L3A21M1, *L. rhamnosus* FB1, *L. paracasei* FB2, *L. paracasei* FB3, and *L. gasseri* BM8. All LABs were previously cultivated in MRS broth at 37 °C. After 48 h of incubation at 37 °C, growth was quantified by measuring the optical density at 600 nm.

### 2.6. Effects of EPS on Fecal Microbiome

#### 2.6.1. Gut Nutrient Medium

In order to cultivate the diverse microbial communities in the gut, it is necessary to provide them with a nutrient-rich environment, such as the gut nutrient medium (GNM) previously described [22,23]. The composition of the GNM was adapted to contain the EPS: 1 g/L EPS, 2 g/L pectin, 1 g/L xylan, 3 g/L potato starch, 0.4 g/L glucose, 3 g/L yeast extract, 1 g/L peptone, 4/L mucin, and 0.5 g/L L-cysteine. This solution was sterilized at 121 °C for 21 min. In addition, a pancreatic juice was prepared with 12 g/L NaHCO_3_, 6 g/L dehydrated oxgall (fresh bile), and 0.9 g/L porcine pancreatin.

#### 2.6.2. Fecal Slurry

In this study, 3 independent experiments were conducted with stool samples from 1-year-old infants (3 collections of individual fecal samples). The selection of infants was based on a diverse microbiota, including the *Bifidobacteriaceae*, *Lactobacillaceae*, and *Enterobacteriaceae* families. Fresh stool samples were collected according to a procedure approved by the Ethics Committee of the University of the Azores (reference number CE/ALN/PT/2022/036). This procedure required parents to sign a consent form on behalf of their children to provide their stool sample. The selection criteria for the donor samples used here were as follows: no intake of antibiotics in the last 3 months, no gastrointestinal disorders, and no intake of probiotics.

At the beginning of the experiment, a 20% (*w*/*v*) fecal slurry was prepared as previously described by Tamargo et al. [23]. Fresh feces were collected under anaerobic conditions. A phosphate saline buffer (0.1 M, pH 7) was used to dilute the sample, and this mixture was then thoroughly mixed with a stomacher (Stomacher 400 Circulater, Seward, UK) at a speed of 230 rpm for 3 min. Each bioreactor was initially filled with 135 mL of the gut nutrient medium (GNM) as previously described by Machado et al. [24]. One reactor, which served as a negative control, contained 135 mL of GNM without EPS. Subsequently, 15 mL of freshly prepared fecal slurry was added to each reactor, resulting in a final volume of 150 mL. The mixture was kept anaerobic and incubated at 37 °C. Samples were taken after 0, 6, 24, and 48 h and analyzed immediately. The pH values of the samples during simulated fermentation were determined by pH meter.

#### 2.6.3. Microbiological Analysis

Decimal dilutions were performed and plated on selective media: PCA for total aerobes, MRS agar for LAB, MRS agar supplemented with L-cysteine 0.5 g/L, raffinose 1 g/L, and lithium chloride 0.05 g/L for bifidobacteria and VRBG for *Enterobacteriaceae* [23,25,26]. The plates were kept in anaerobiosis at 37 °C for 24 to 48 h, except for the total aerobic count (PCA). Colony counts on all plates were performed in duplicate and the results are expressed as colony forming units per milliliter (CFU/mL).

### 2.7. Statistical Analysis

All experiments were performed in triplicate and the results are expressed as mean ± SEM. Analysis of variance (one-way ANOVA) was used to evaluate the effect of the treatments on the different parameters studied. The Tukey test was performed to detect significant differences (*p* < 0.05). Statistical analysis was performed using SPSS software, version 27 (SPSS, Chicago, IL, USA).

## 3. Results and Discussion

### 3.1. Biological Activities of EPS Produced by Ln. Mesenteroides SJC113

#### 3.1.1. Cholesterol-Lowering Ability

EPS showed a strong cholesterol-binding effect and effectively reduced cholesterol levels in cholesterol-enriched MRS broth (Table 2). A 0.1% EPS concentration reduced cholesterol levels by over 40%, while a 0.01% EPS concentration achieved a reduction of 33.01 ± 2.78%. These results are in agreement with the findings of several authors who reported similar levels of cholesterol-binding of heteropolysaccharides produced by *Limosilactobacillus fermentum* and *Lactiplantibacillus plantarum* strains [17,27,28,29]. To our knowledge, there are no previous reports describing the cholesterol-binding ability of α-glucan-type EPS or EPS produced by *Leuconostoc* spp. However, several studies have reported the cholesterol-lowering effect of *Leuconostoc* spp. [30,31,32]. The cholesterol-binding capacity of EPS may play a key role in reducing intestinal cholesterol absorption, thereby improving cardiovascular health. Recent studies highlight the hypocholesterolemic effect of EPS-producing strains (especially *Lactobacillus* and *Leuconostoc mesenteroides*) in vivo by assimilating cholesterol and excreting it via feces [33]. Therefore, the inclusion of these EPS in food formulations could be a useful approach for the production of functional foods with cholesterol-lowering potential.

#### 3.1.2. Antioxidant Activity

The EPS produced by *Ln. mesenteroides* SJC113 showed moderate antioxidant activity with scavenging rates of DPPH radicals ranging from 10.94 ± 1.33% to 17.48 ± 1.74% at concentrations in the range of 1–3 mg/mL (Table 2). This activity was lower than the values reported by Amiri et al. [34], where various LAB strains achieved up to 62.33 ± 1.02% radical scavenging at 2 mg/mL EPS, and was significantly lower than the 86.2% activity reported by Hu et al. [18] for EPS-2 at 5 mg/mL. The hydroxyl radical scavenging activity was also modest (6.29 ± 1.59% to 9.34 ± 1.89% at 1–3 mg/mL), in contrast to the activity (59.94%) reported by Amiri et al. [34] for EPS produced by a *Lactobacillus acidophilus* strain. However, other authors also reported reduced antioxidant activity (both DPPH scavenging activity and hydroxyl scavenging activity) for bacterial EPS < 4 mg/mL [35].

Studies on the antioxidant activity of EPS show that it does not correlate directly with the amount of EPS, but depends on both the bacterial strain and the culture conditions [36,37]. These results emphasize that the antioxidant potential of EPSs varies greatly depending on the LAB strains and is strongly influenced by its composition. Huang et al. [38] demonstrated that the antioxidant effect of purified EPS is primarily due to the protein components rather than the polysaccharide components, emphasizing the importance of protein-associated mechanisms. In addition, the molecular weight (MW) of EPS has a significant influence on its bioactivity [39]. Hydroxyl groups, which serve as important free radical scavengers, are less accessible in polysaccharides with high MW, which impairs their antioxidant efficacy. The high viscosity and steric hindrance in larger EPS molecules further limit mobility and interactions with radicals in solution. The lower antioxidant performance of EPS produced by *Ln. mesenteroides* SJC113 may therefore be due to a combination of high molecular weight, limited exposure of hydroxyl groups, and low protein content of the purified sample.

### 3.2. Prebiotic Properties

#### 3.2.1. Adhesion to Immobilized Mucin

Table 3 presents the mucin-binding capacity of *Ln. mesenteroides* SJC113, evaluated alongside the probiotic reference strain *L. rhamnosus* GG. Mucin adhesion is a critical determinant of probiotic functionality, as it enhances gut colonization potential and host-microbe interactions. *Ln. mesenteroides* SJC113 demonstrated high adhesion levels (74.1%) comparable to *L. rhamnosus* GG (75.5%), suggesting strong probiotic potential for this strain.

#### 3.2.2. Adhesion to Mucin in the Presence of EPS

**Figure 1:**
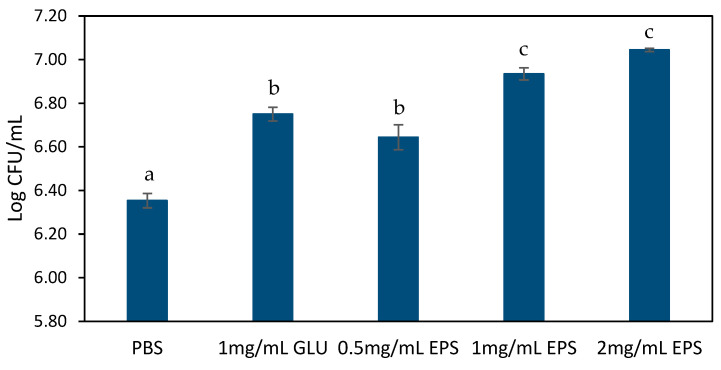
Mucin adhesion of *L. rhamnosus* GG in the presence of EPS or glucose. The values are the average of three experiments ± SEM. Different letters (a–c) indicate significant differences (*p* < 0.05) between treatments.

The role of the EPS in modulating probiotic adhesion to human intestinal mucus was also investigated in this study. Using *L. rhamnosus* GG as a probiotic model strain, we assessed the mucin binding capacity in the presence of different EPS concentrations (0.5–2 mg/mL). As shown in Figure 1, EPS significantly (*p* < 0.05) enhanced the adhesion of *L. rhamnosus* GG to mucin in a dose-dependent manner. The maximal adhesion of 5.6 × 10^6^ and 1.1 × 10^7^ CFU/mL occurred at 1 and 2 mg/mL EPS, respectively, and was significantly higher (*p* < 0.05) than the positive control (1 mg/mL of glucose) and negative control (PBS). The 7.04 Log CFU/mL adhesion at 2 mg/mL exceeds values reported for *L. rhamnosus* GG in the EPS-free system (PBS) in the present study and by other authors (typically 5.8–6.2 Log CFU/mL) [40,41], indicating synergistic effects of the EPS in adhesion to mucin. These results suggest that EPS may serve as an important mediator of probiotic-mucin interactions, possibly through surface modification or direct binding facilitation.

#### 3.2.3. Stimulation of LAB Growth

**Figure 2:**
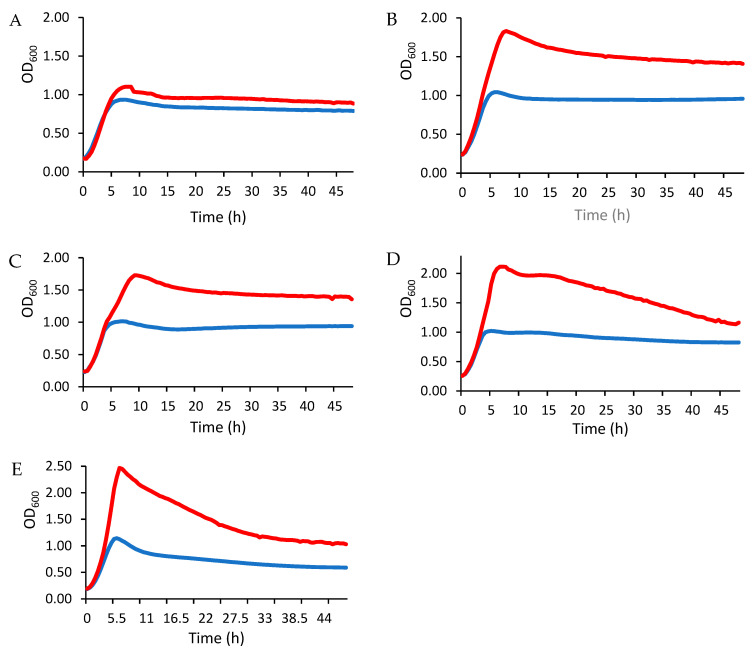
Growth of LAB evaluated by OD at 600 nm in MRS medium supplemented with glucose only (blue line) or with glucose and EPS (red line). LAB strains: (**A**) *L. lactis L3A21M1*, (**B**) *L. paracasei* FB2, (**C**) *L. paracasei* FB3, (**D**) *L. rhamnosus* FB1, and (**E**) *L. gasseri* BM8.

The changes in LAB growth in the presence of EPS are shown in Figure 2. Overall, the exponential growth phase—characterized by a rapid increase in optical density (OD)—was significantly more pronounced in the EPS-supplied cultures than in the untreated controls. The results also indicate a strain-dependent enhancement of LAB growth after EPS treatment, with the effect being more pronounced in lactobacilli (Figure 2B–E) than in *Lactococcus* (Figure 2A). The sustained increase in OD values in the EPS-treated groups indicates a direct stimulatory effect of EPS on LAB growth and emphasizes its potential as a microbial growth promoter.

The observations in the present study are consistent with previous reports by Zhao et al. [42], which showed that LAB cultured in MRS medium supplemented with both glucose and dextran or commercial prebiotics exhibited significantly better growth kinetics than LAB in glucose-only medium. In addition, these authors reported that the effect of prebiotics on different probiotic bacteria was not only strain-dependent but also depended on the type of polysaccharides. These results suggest that EPS-mediated growth promotion may be a generalizable phenomenon that depends on the EPS type and the LAB strain. Although reference prebiotics, such as inulin, FOS, or GOS, were not tested in the present study, several studies reported that carbohydrates with longer chain lengths are fermented more slowly by LAB strains [43]. For example, inulin showed limited growth stimulation of *L. casei* strains compared to FOS [44]. Further investigation of the strain-specific mechanisms underlying EPS utilization could provide valuable insights into the optimization of probiotic production and functional food applications.

The measurement of OD (at 600 nm) was employed as the primary growth assessment method, providing real-time, non-destructive monitoring of bacterial strain dynamics under EPS exposure. While this method captures total biomass accumulation, its correlation with cellular growth remains robust in controlled monocultures, making it ideal for comparative analysis of EPS-mediated growth patterns across LAB strains [43,45,46]. However, for specific investigations of viability cell quantification under EPS treatment, complementary plating assays (CFU) should be included.

#### 3.2.4. Effects of EPS on Selected Bacterial Groups in Fecal Fermentation

Adhesion to intestinal mucus is the first step of microbial colonization of the gut and plays a crucial role in modulating immune function and improving the integrity of the intestinal barrier. Despite their importance, the influence of dietary polysaccharides on the gut microbiota of infants is still poorly understood. Dextrans that resist digestion can promote beneficial bacteria such as *Bifidobacterium*—a dominant microbial genus in infants that plays a key role in establishing a healthy infant gut microbiota health [47].

**Figure 3:**
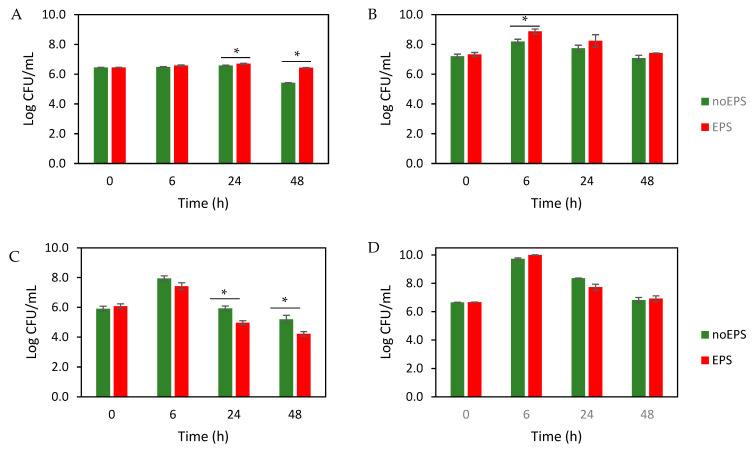
Effect of EPS on microbial growth (Log CFU/mL) of (**A**) *Bifidobacterium*, (**B**) LAB, (**C**) *Enterobacteriaceae*, and (**D**) total aerobes. The values are the average of three experiments ± SEM. * Indicates significant difference at *p* < 0.05.

In this study, the prebiotic potential of a dextran-like EPS using infant stool samples were investigated, focusing on *Bifidobacterium* spp. and LAB. The number of bacteria over time (Figure 3) showed that EPS significantly increased *Bifidobacterium* spp. after 24 h and 48 h (*p* < 0.05; Figure 3A) and LAB after 6 h (*p* < 0.05; Figure 3B). In contrast, EPS reduced *Enterobacteriaceae* after 24 h and 48 h (*p* < 0.05; Figure 3C), with no significant effect on the total number of aerobes (*p* > 0.05; Figure 3D). These results are consistent with previous reports showing the stimulatory effect of dextran on the growth of bifidobacteria and lactobacilli [48].

In the present study, selective plate cultures were used to evaluate the effects of EPS on the fecal microbiota in vitro. While the use of selective media is an established approach to evaluate specific bacterial groups, this method is not without limitations. Selective media can lead to false-positive results and a lack of resolution at the species level, which may not fully capture the actual growth dynamics of the target bacterial species. Therefore, the accuracy of the results should be interpreted with caution considering this methodological limitation. For accurate profiling, molecular methods (16S rRNA sequencing, qPCR) or colony validation (MALDI-TOF MS) should complement traditional cultivation to account for media-related biases. This analysis should be conducted in further follow-up studies.

**Figure 4:**
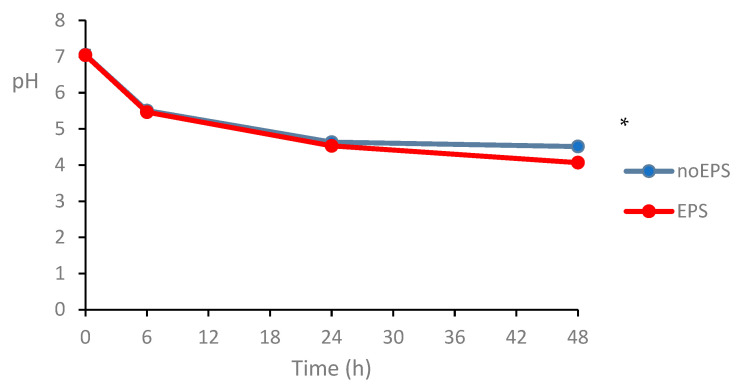
Effect of EPS on pH of fecal slurry over 48 h incubation at 37 °C. * Indicates significant difference at *p* < 0.05.

A notable observation was the significant pH reduction during 48 h fermentation (*p* < 0.05; Figure 4), likely attributable to microbial production of acidic metabolites, such as short-chain fatty acids (SCFAs). Although SCFA quantification was not performed in this study, the pH trend is consistent with previous reports in which EPS fermentation by the gut microbiota resulted in increased acetate and propionate levels [49,50]. However, the lack of direct SCFA measurements limits the ability to conclusively link the pH drop to specific metabolic pathways, highlighting an important area for further investigation.

The results of the present work support the existing evidence that certain EPS exhibit selective prebiotic effects, stimulating beneficial microbes while suppressing pathogens in the human gut [51,52]. For example, EPS produced by *Lacticaseibacillus paracasei* and *Lactobacillus sanfranciscensis* exhibit similar bifidogenic properties while reducing pathogenic bacteria (*Escherichia-Shigella, Klebsiella*, and *Fusobacterium*) [51,52]. In contrast, some EPSs, such as those from *Lactobacillus rhamnosus* RW-9595M, show minimal modulation of infant microbiota [53], underscoring the structure-dependent variability in prebiotic activity.

In the present study, the results indicate that EPSs of *Ln. mesenteroides* SJC113 can selectively promote *Bifidobacterium* while inhibiting *Enterobacteriaceae*, which strengthens its potential as a targeted prebiotic. These observations emphasize the intricate relationship between EPS structure and the modulation of the infant gut microbiota, and reinforce the potential of EPS produced by this strain as an effective prebiotic. However, the translational relevance of these in vitro observations remains to be validated in more complex systems, such as in vivo infant models or human trials.

While this study provides insights into the prebiotic potential of EPS from *Ln. mesenteroides* SJC113, several limitations must be acknowledged. First, the observed antioxidant activity of this EPS was modest compared to other reported EPSs, suggesting that its primary benefits may lie in microbial modulation rather than direct free radical scavenging. Secondly, like all in vitro models, the fecal fermentation system cannot fully replicate the physiological complexity of the infant gut, where host factors (e.g., immune responses, mucosal interactions, and nutrition) significantly influence microbial dynamics. Although selective plate counting allows the targeted analysis of key bacterial groups, this approach may miss subtle shifts in the community or functional changes that can only be revealed by metagenomic or metabolomic methods. Finally, the present experiments were based on three individual donor samples, which avoid the use of artificially pooled stool samples, but may limit the generalizability of the results, given the large inter-individual variability in the composition of the gut microbiota of infants. Future work should investigate the inter-individual variability and long-term effects to better assess the therapeutic potential of this EPS in infant feeding.

## 4. Conclusions

This study comprehensively characterized the multifunctional properties of the EPS produced by *Ln. mesenteroides* SJC113, revealing its promising prebiotic potential. The EPS exhibited moderate but consistent antioxidant activity, effectively scavenging both DPPH and hydroxyl radicals in dose-dependent assays. More notably, the EPS displayed a pronounced cholesterol-lowering effect, reducing cholesterol levels in the culture medium by 40%. The EPS also exhibited noteworthy prebiotic potential, demonstrating dual functionality in microbial adhesion and growth promotion. While *Ln. mesenteroides* SJC113 displayed strong intrinsic mucin-binding capacity, its EPS further enhanced the adhesion of the probiotic *L. rhamnosus* GG to mucin—a critical trait for gut colonization and host interaction. Beyond adhesion, the EPS served as an effective fermentable substrate, consistently stimulating the growth of several LAB strains. In gut-mimicking conditions, the EPS selectively modulated bacterial populations, significantly increasing LAB and *Bifidobacterium* spp. while suppressing *Enterobacteriaceae,* thereby validating its prebiotic efficacy. These multifunctional properties position the EPS-producing *Ln. mesenteroides* SJC113 as a versatile candidate for food and pharmaceutical applications, offering a novel strategy to engineer functional foods and targeted therapies for gut health enhancement. Future research should focus on elucidating the molecular mechanisms—in particular, mucus adhesion and the cholesterol-binding of EPS—using advanced models (e.g., mucus-secreting cell lines and omics approaches) and validating its efficacy using in vivo models to fully exploit the potential of this EPS for human health.

## Figures and Tables

**Table 1:** Bacterial strains used in the present study.

Strains	Origin	ATCC/GenBank Accession Number	Source
*Lacticaseibacillus rhamnosus* GG	Human gut	ATCC 53103	Dicofarm
*Leuconostoc mesenteroides* SJC113	Cheese	MT742947	IITAA Collection
*Lactococcus lactis* L3A21M1	Cheese	KF193424	IITAA Collection
*Lacticaseibacillus rhamnosus* FB1	Human feces	OQ713701	IITAA Collection
*Lacticaseibacillus paracasei* FB2	Human feces	OQ713702	IITAA Collection
*Lacticaseibacillus paracasei* FB3	Human feces	OQ713703	IITAA Collection
*Lactobacillus gasseri* BM8	Human milk	OQ713687	IITAA Collection

A hyphen (-) indicates that testing for that EPS concentration was not performed. Different letters as superscripts in the same line indicate a significant difference (*p* < 0.05).

**Table 2:** Cholesterol-binding ability (%), radical scavenging activity—DPPH (% and equivalent concentration of ascorbic acid (mg/mL)), and hydroxyl scavenging activity (% and equivalent concentration of ascorbic acid (mg/mL)) of EPS produced by *Ln. mesenteroides* SJC113. Ascorbic acid (1 mg/mL) is presented as the positive control. Results are presented as mean ± SEM (n = 3).

EPS *	0.1 mg/mL	1 mg/mL	2 mg/mL	3 mg/mL
Cholesterol-binding (%)	33.01 ± 2.86 ^a^	40.18 ± 2.42 ^b^	-	-
Radical scavenging activity—DPPH (%)	-	10.94 ± 1.03 ^a^	13.15 ± 0.81 ^ab^	17.48 ± 1.74 ^b^
Ascorbic acid (%)	-	89.3 ± 4.8	-	-
Eq. ascorbic acid (mg/mL)	-	0.092 ± 0.017 ^a^	0.119 ± 0.011 ^ab^	0.164 ± 0.015 ^b^
Hydroxyl scavenging activity (%)	-	6.29 ± 1.46 ^a^	8.57 ± 2.29 ^a^	9.34 ± 2.06 ^a^
Ascorbic acid (%)	-	65.0 ± 1.2	-	-
Eq. ascorbic acid (mg/mL)	-	0.065 ± 0.024 ^a^	0.111 ± 0.039 ^a^	0.128 ± 0.033 ^a^

* A hyphen (-) indicates that testing for that EPS concentration was not performed. Different letters as superscripts in the same line indicate a significant difference (*p* < 0.05).

**Table 3:** LAB adhesion to immobilized mucin expressed as viable counts (Log CFU/mL) after 90 min of incubation. Values are the average of three independent experiments ± SEM.

Strain	Log CFU/mL	
	0 min *	90 min
*Ln. mesenteroides* SJC113	11.64 ± 0.07	8.62 ± 0.08
*L. rhamnosus* GG	10.63 ± 0.06	8.03 ± 0.25

* Initial counts.

## Data Availability

The original contributions presented in the study are included in the article, further inquiries can be directed to the corresponding author.
